# Updating the Molecular Spectrum of Non-Deletional Hb H and Hb H-like Disease in Southern Thailand

**DOI:** 10.3390/ijms27219970

**Published:** 2026-11-06

**Authors:** Wanicha Tepakhan, Sitthichai Panyasai, Wittaya Jomoui, Sataron Kanjanaopas, Korntip Srewaradachpisal, Pornpun Sripornsawan

**Affiliations:** 1 Centre for Research and Development of Medical Diagnostic Laboratories, Faculty of Associated Medical Sciences, Khon Kaen University, Khon Kaen 40002, Thailand; 2 Department of Pathology, Faculty of Medicine, Prince of Songkla University, Songkhla 90110, Thailand; 3 Department of Medical Technology, School of Allied Health Sciences, University of Phayao, Phayao 56000, Thailand; 4 Department of Pathology, Maha Chakri Sirindhorn Medical Center, Faculty of Medicine, Srinakharinwirot University, Nakhon Nayok 26120, Thailand; 5 Medical Genetic and Genomics Laboratory Unit, Maha Chakri Sirindhorn Medical Center, Faculty of Medicine, Srinakharinwirot University, Nakhon Nayok 26120, Thailand; 6 Research Cluster in Hematology and Genetic Diseases, Faculty of Medicine, Srinakharinwirot University, Nakhon Nayok 26120, Thailand; 7 Department of Pediatrics, Faculty of Medicine, Prince of Songkla University, Songkhla 90110, Thailand

**Keywords:** molecular spectrum, non-deletional Hb H disease, Hb H-like disease, Thailand

## Abstract

Non-deletional hemoglobin (Hb) H and Hb H-like diseases are clinically significant forms of α-thalassemia with variable severity. In this study, we aimed to update the molecular spectrum of these conditions in southern Thailand and revise the allele proportions of α-thalassemia mutations among screened carriers from different regions in Thailand. A total of 118 patients with non-deletional Hb H or Hb H-like disease were characterized using DNA sequencing and multiplex ligation-dependent probe amplification analysis, and 679 possible α-thalassemia carriers were analyzed to determine allele proportion. Ten distinct genotypes were identified. Hb H-Constant Spring (CS) disease was the most frequent (79.7%), followed by non-deletional Hb H with Hb E heterozygosity (13.6%). Among patients with non-deletional Hb H disease, 76.5% of those with Hb H-CS disease and 68.8% of those with non-deletional Hb H disease and Hb E heterozygosity were classified as NTDT. Three cases of Hb H-like disease with a TDT phenotype caused by compound heterozygous Hb CS/Hb Adana were identified for the first time in Thailand. Homozygous Hb Chumphae was also reported for the first time to exhibit a mild clinical phenotype. Hb Adana was detected at an allele proportion of 1.8% among screened carriers from Southern Thailand, and a multiplex AS-PCR for simultaneous detection of Hb CS, Hb Paksé, and Hb Adana was developed.

## 1. Introduction

Alpha (α) thalassemia is an inherited disorder of the α-globin (*HBA*) gene, resulting in decreased (α^+^-thalassemia) or lack of production (α^0^-thalassemia) of the α-globin chain. Large deletions of one *HBA* gene (–α), such as –α^3.7^ (NG_000006.1:g.34164_37967del3804) and –α^4.2^ (NC_000016.10:g.169818_174075del), through unequal crossing over in the *HBA* gene cluster constitute the common defect in α^+^-thalassemia. However, some point mutations cause abnormal α-globin chain structure and decreased α-globin production, leading to non-deletional α-thalassemia (α^T^-thalassemia), such as hemoglobin (Hb) Constant Spring (CS, *HBA2*:c.427T>C) and Hb Paksé (PS, *HBA2*:c.429A>T) [1]. These genetic defects are commonly reported in Southeast Asian populations with diverse ethnic backgrounds [2]. In Thailand, the prevalence of α-thalassemia carriers is 30–40%, and the common α-thalassemia defects include α^0^-thalassemia (– –SEA (NC_000016.10:g.165397_184700) and – –THAI (NC_000016.10:g.149863_183312)), α^+^-thalassemia (–α^3.7^, –α^4.2^), Hb CS, and Hb PS [3].

Homozygous α^0^-thalassemia (– –/ – –) is the most severe type of α-thalassemia and results in Hb Bart’s hydrops fetalis. In addition, a compound heterozygous α^0^/α^+^-thalassemia mutation that affects three *HBA* genes, resulting in extremely low α-globin chain production, causes Hb H disease. Consequently, the excess β-globin chains aggregate into Hb H tetramers, which are unstable hemoglobins that precipitate within red blood cells, leading to hemolytic anemia. Patients with compound heterozygous α^0^-thalassemia and deletional α^+^-thalassemia (– –/–α), referred to deletional Hb H disease, present with a milder phenotype mostly non-transfusion-dependent thalassemia (NTDT). However, a hemolytic crisis may occur in the presence of a concomitant illness that increases oxidative stress. In contrast, non-deletional Hb H disease is attributed to the interaction of α^0^-thalassemia with α^T^-thalassemia (– –/α^T^α). Non-deletional Hb H disease has a more severe clinical phenotype and is sometimes classified as transfusion-dependent thalassemia (TDT) [4]. Homozygosity or compound heterozygosity of α^T^-thalassemia (α^T^α/α^T^α) can also lead to severe clinical manifestations, such as transfusion-dependent Hb H-like disease, or hydrops fetalis [5,6]. Thus, the heterogeneity of α-thalassemia mutations in the Southeast Asian population could result in a wide varieties of interactions and clinical phenotypes.

Thalassemia carriers are generally asymptomatic; however, at-risk couples may have offspring with severe disease due to the autosomal recessive inheritance pattern. Accordingly, thalassemia screening is an essential public health measure [7]. In Thailand, severe thalassemia occurs in approximately 1 in 100 births [8]. The national thalassemia prevention and control program has reduced the incidence of Hb Bart’s hydrops fetalis and severe β-thalassemia [9]. However, routine molecular diagnosis solely focuses on α^0^-thalassemia to prevent Hb Bart’s hydrops fetalis. As a result, the incidence of transfusion-dependent Hb H disease, primarily non-deletional α^T^-thalassemia, has increased significantly in Thailand. Approximately 3000 newborns were diagnosed with Hb H disease in 2020 [8]. Moreover, fetal hydrops associated with non-deletional α^T^-thalassemia requiring intrauterine transfusion has also been reported [10,11].

Recently, we reported the genotype frequencies and hematological profiles of common and rare Hb H diseases in southern Thailand. Non-deletional Hb H disease was reported in 27% of cases. Unusual forms of Hb H disease were observed in approximately 5.8% of cases and were caused by rare α^0^-thalassemia mutations (– –SA (NG_000006.1:g.19464_43064del23601) or – –CR (NC_000016.10:g.144,215_188,841)) [12]. However, some cases of non-deletional Hb H disease or Hb H-like disease caused by an unknown point mutation in the *HBA* gene remain to be characterized. Thus, in this study, we aimed to update the molecular spectrum of non-deletional Hb H and Hb H-like disease in southern Thailand and to revise the current allele frequency of α-thalassemia mutations in the Thai population.

## 2. Results

### 2.1. Identification of Genotypes in Patients with Non-Deletional Hb H or Hb H-like Disease

**Figure 1:**
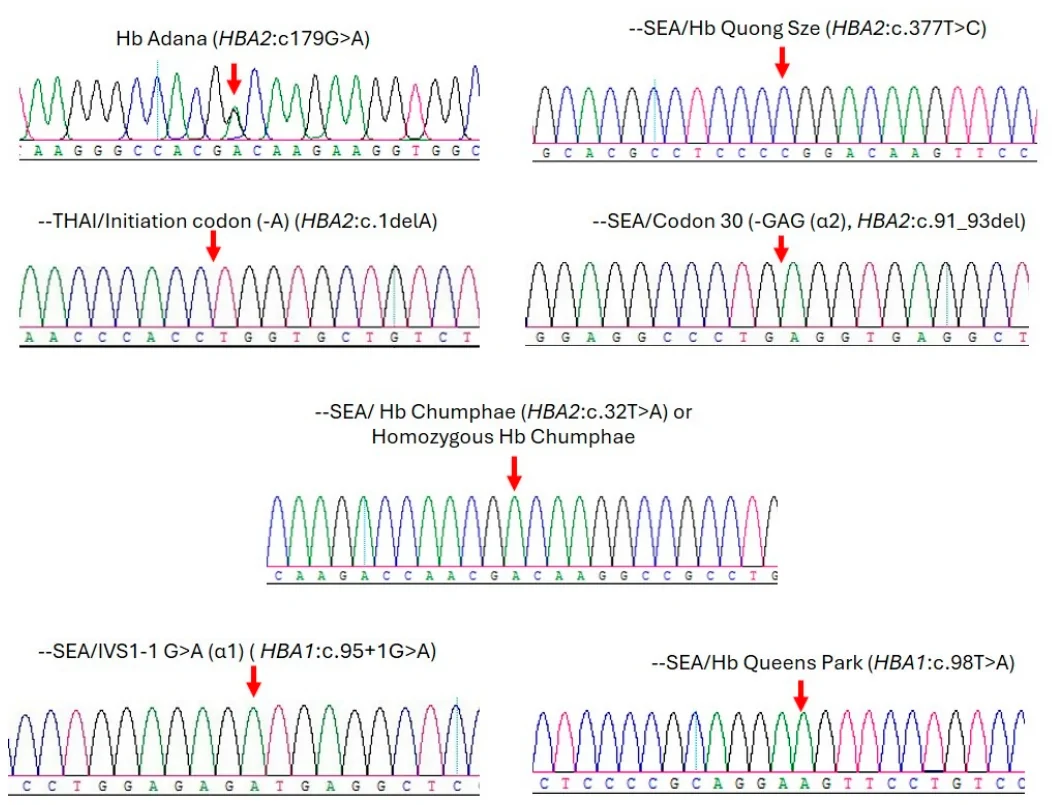
DNA sequencing results for patients with uncommon non-deletional Hb H and Hb H-like disease. Abbreviation: Hb, hemoglobin.

**Figure 2:**
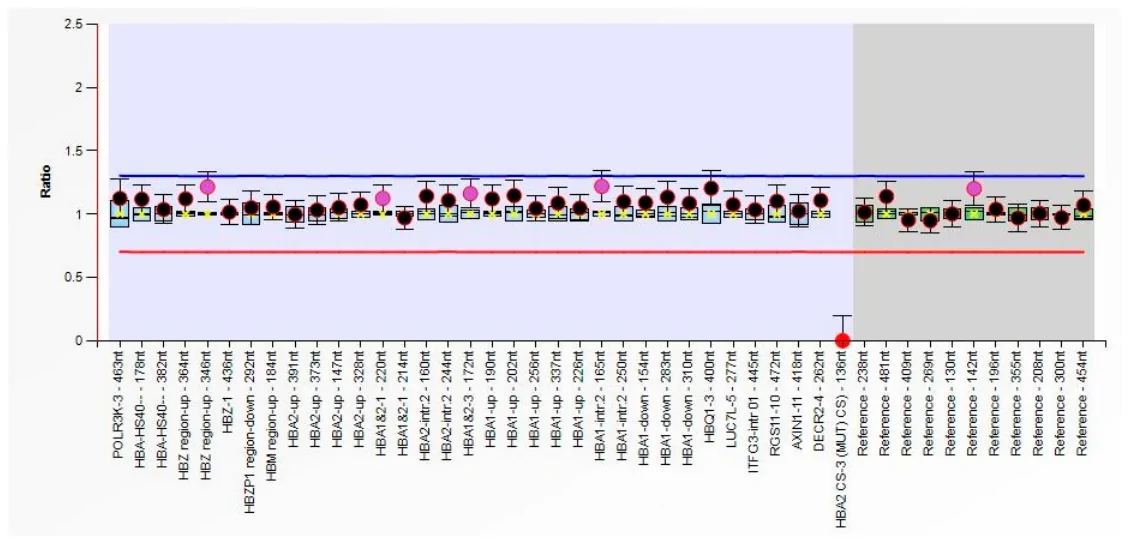
MLPA results for homozygous Hb Chumphae without deletional mutation in the *HBA* cluster. MLPA results showing the fluorescence ratios of the patient′s sample compared with the reference samples. The ratios are within the normal range (0.7–1.3), confirming the absence of gene deletion in the patient. Abbreviations: Hb, hemoglobin; MLPA, multiplex ligation-dependent probe amplification.

Among the 118 patients, 10 cases remained uncharacterized and were categorized as non-deletional Hb H or Hb H-like disease. Subsequent DNA sequencing identified three cases of Hb Adana (*HBA2*:c.179G>A) co-inherited with Hb CS. The remaining cases carried α^0^-thalassemia (– –SEA or – –THAI) in combination with rare α^T^-thalassemia variants. These included Hb Quong Sze (Hb QS, *HBA2*:c.377T>C), Hb Chumphae (*HBA2*:c.32T>A), an initiation-codon (-A) (*HBA2*:c.1delA), and a codon30 (-GAG, *HBA2*:c.91_93del) in the *HBA2* gene, along with the IVS1-1 (G>A, *HBA1*:c.95+1G>A) and Hb Queens Park (*HBA1*:c.98T>A) in the *HBA1* gene. One patient carried homozygous Hb Chumphae in the absence of any α^0^-thalassemia mutation (Figure 1). This finding was further validated via multiplex ligation-dependent probe amplification (MLPA) analysis, which ruled out any deletional mutation within the *HBA* cluster (Figure 2). The molecular characteristics of eight α^T^-thalassemia variants, their functional effects or stability are summarized in Table 1.

### 2.2. Genotype Frequency and Hematological Manifestations of Non-Deletional Hb H or Hb H-like Disease

The genotype spectrum and hematological parameters of non-deletional Hb H or Hb H-like disease are summarized in Table 2. Ten distinct genotypes were identified among the 118 patients. The most frequent genotype was Hb H-CS disease, accounting for 94 patients (79.7%). Sixteen patients (13.6%) carried non-deletional Hb H disease in combination with Hb E heterozygosity, among whom 14 had Hb H-CS, 1 had Hb H-Quong Sze, and 1 had Hb H-α1 IVS1-1 (G>A). Four patients presented with rare genotypes, namely Hb H-α2 codon 30 (-GAG), Hb H-Queens Park, Hb H-α2 initiation codon (-A), and Hb H-Chumphae.

Non-deletional Hb H disease attributed to a point mutation in *HBA1*, including Hb H-α1 IVS1-1 (G>A) and Hb H-Queens Park, presented with less severity than that caused by mutations in the *HBA2* gene, such as Hb H-CS, Hb H-α2 codon 30 (-GAG), and Hb H-α2 initiation codon (-A). Patients with non-deletional Hb H disease and Hb E heterozygosity presented with lower Hb E levels (9.4–15.3%), no detectable Hb H, and slightly increased Hb Bart’s levels. Three patients with compound heterozygous of Hb CS and Hb Adana exhibited thalassemia intermedia to severe hematological phenotypes (median Hb 7.2, interquartile range [IQR] 6.7–8.7 g/dL) and all of them had a history of blood transfusion. A patient with Hb H-Chumphae presented with neonatal anemia and jaundice requiring phototherapy, which was resolved later. After infancy, this patient had only mild asymptomatic anemia (Hb 10.1 g/dL), normal growth, and no history of blood transfusion. Another patient with homozygous Hb Chumphae also presented with mild clinical symptoms (Hb 10.6 g/dL), and inclusion bodies were positive without detectable Hb H levels. However, patients with Hb H-Chumphae had higher red cell distribution width (33.7 vs. 23%) and Hb Bart’s levels (25.9% vs. 5.5%) compared with patients homozygous for Hb Chumphae (Table 2).

The proportion of NTDT phenotypes in patients with non-deletional Hb H disease was not significantly different from those patients with non-deletional Hb H disease combined with the Hb E heterozygosity (76.5% and 68.8%, respectively). However, MCV (median 56.0, IQR 54.0–60.0 fL) and mean corpuscular hemoglobin (MCH) (median 16.7, IQR 15.6–17.2 pg) values were significantly lower in patients with non-deletional Hb H disease with the Hb E heterozygosity than in those with non-deletional Hb H disease alone (*p* < 0.001) (Table 3).

### 2.3. Updating the Allele Proportions of α-Thalassemia Mutations Among Screened Carriers from Different Centers in Thailand

Molecular screening revealed that 525 (77.3%) of the 679 possible α-thalassemia carriers based on first-level hematology screening carried α-thalassemia mutations. Hb Adana was newly noticed with an allele proportion of 1.8% in the southern regions. The – –SEA mutation was predominant in northern Thailand (62.5%), while the –α^3.7^ mutation was more common in central/eastern and southern regions (60.9% and 52.1%, respectively). Hb CS was the most prevalent non-deletional α^T^-thalassemia variant in northern Thailand (8.9%) but was occasionally found in central/eastern (2.6%) and southern (3.1%) Thailand (Table 4).

**Figure 3:**
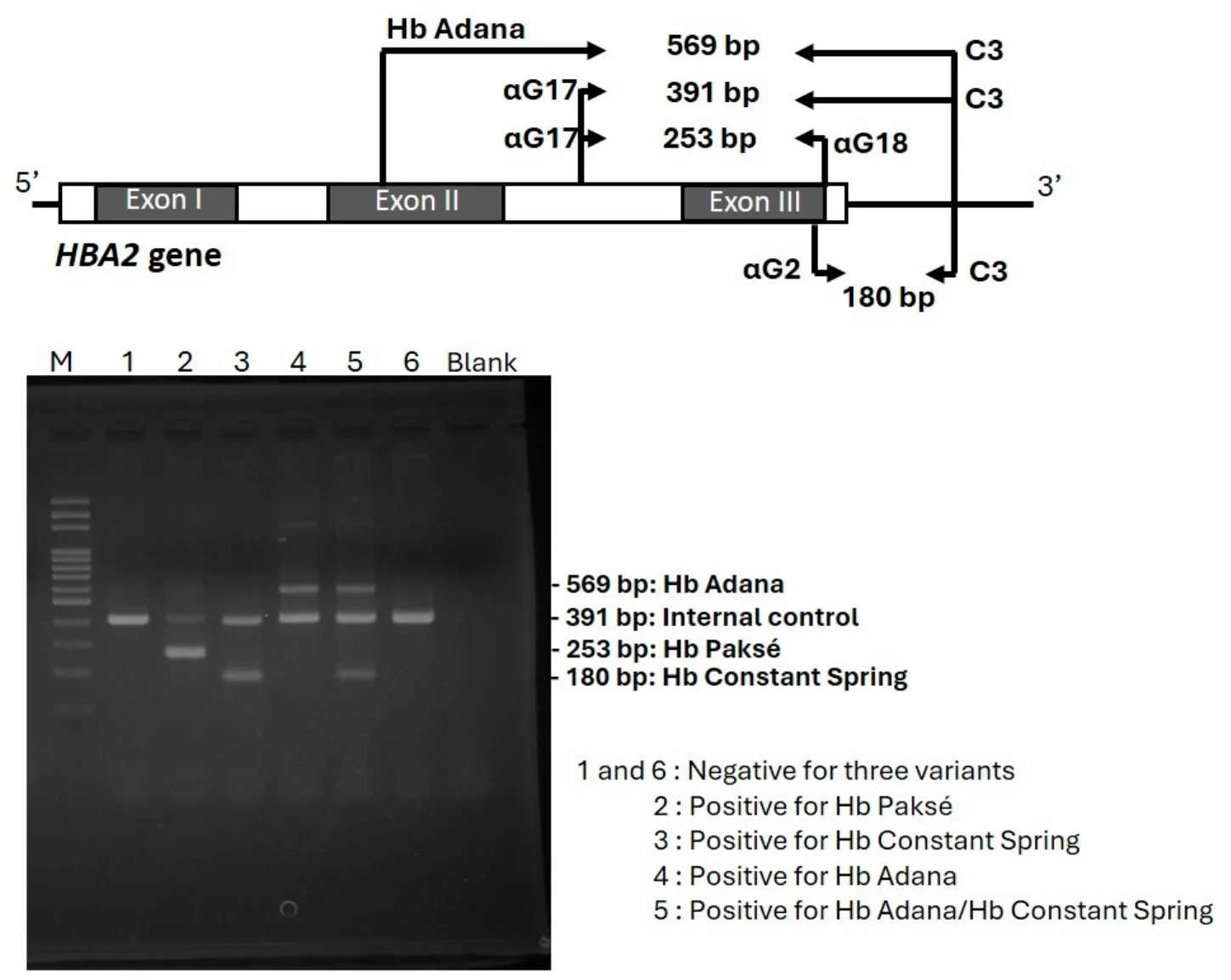
Schematic diagram of primer orientation and agarose gel electrophoresis results for a newly developed multiplex AS-PCR for Hb Constant Spring, Hb Paksé, and Hb Adana. Abbreviations: AS-PCR, allele-specific polymerase chain reaction; Hb, hemoglobin.

The presence of Hb Adana in southern populations informed the development of a multiplex AS-PCR for prompt identification of non-deletional α^T^-thalassemia variants, including Hb CS, Hb PS, and Hb Adana, simultaneously in one tube (Figure 3).

## 3. Discussion

The high frequency of thalassemia alleles in Thailand and Southeast Asian countries is widely believed to have resulted from natural selection associated with protection against malaria [13]. In Thailand, Hb H disease is commonly encountered, with deletional forms accounting for the majority of cases (71.6%) and non-deletional forms comprising the remaining 28.4% [14]. However, the frequency of specific Hb H disease genotypes differs across Thailand. Non-deletional Hb H disease is more frequent among patients from central (51.6%) and northern (53.9%) regions [15,16]. whereas deletional Hb H disease predominates in the northeastern (57.3%) and southern (72.2%) regions [12,17]. Further complexity arises from the high prevalence of Hb E in these populations, as its coinheritance with Hb H disease can produce a distinct clinical entity known as EA Bart’s disease. The incidence of EA Bart’s disease also varies by region, with the current study reporting rates of 13.5% in southern Thailand, 30.6% in the northeast, and 25.9% in the central region [12,15], underscoring regional heterogeneity in the distribution of complex hemoglobin disorders across Thailand.

This study revealed substantial genotypic heterogeneity among individuals with non-deletional Hb H disease in southern Thailand, correlating to a broad spectrum of disease severity. Patients presented with mild to severe anemia, characterized by Hb levels ranging from 5.7 to 10.1 g/dL. This variability in clinical presentation is primarily determined by the specific *HBA* gene involved and the type of mutation disrupting α-globin chain production. The *HBA1* gene contributes approximately one-third the α-globin chain output of the *HBA2* gene [18]; therefore, mutations affecting *HBA1*, such as Hb H-α1 IVS1-1 (G>A) and Hb H-Queens Park, result in a relatively milder reduction in α-globin chain production. The residual α-globin chains produced from the remaining functional alleles are sufficient to pair with β-globin chains for Hb A assembly, thereby reducing the pool of unpaired β-globin chains and limiting the formation of β_4_ tetramers (Hb H) responsible for hemolytic anemia. In contrast, mutations affecting the *HBA2* gene, such as Hb H-CS, Hb H-α2 codon 30 (-GAG), and Hb H-α2 initiation codon (-A), have a more profound impact on total α-globin chain output. This raises the number of unpaired β-globin chains, increases Hb H formation, and enhances precipitation of Hb H inclusions within red blood cells, ultimately resulting in more severe hemolytic anemia. Of particular interest was a case of Hb H-Chumphae disease in which the Hb Chumphae mutation occurred in the *HBA2* gene, yet presented with only mild anemia. The Hb level (10.1 g/dL) was comparable to that observed in homozygous Hb Chumphae (10.6 g/dL). The mild clinical severity is likely because Hb Chumphae retains partial function under normal physiological conditions but remains susceptible to oxidation and precipitation under oxidative stress in a manner analogous to that of Hb H. Meanwhile, the intact *HBA1* gene continues to produce normal Hb A, keeping excess β-globin chains at a level too low to form detectable Hb H or exceed the 5% erythrocyte threshold required for detection by CE technique [19]. Recent study demonstrated that Hb Chumphae is an unstable variant. The low level and instability of Hb Chumphae is not due to instability of the hemoglobin tetramer. Rather, it is likely caused by reduced mRNA synthesis or stability, together with rapid proteolysis of the α^Chumphae^-globin chain, in which the substitution of valine with a negatively charged aspartate probably disrupts the α-helix structure [20].

**Figure 4:**
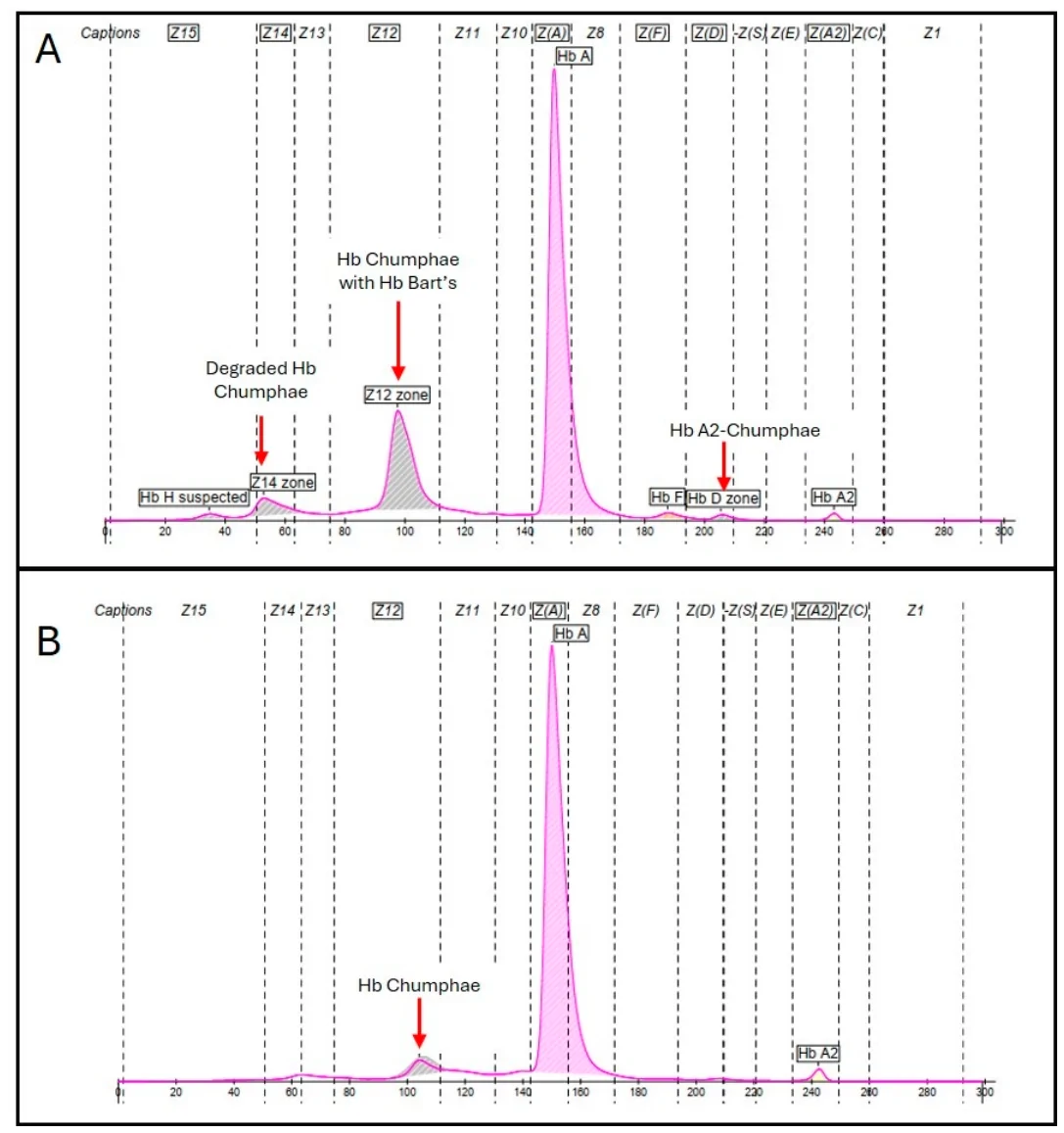
Electropherogram of Hb H-Chumphae (**A**) and homozygous Hb Chumphae (**B**) using capillary electrophoresis. Abbreviations: Hb, hemoglobin.

Hb H-Chumphae was first reported in a neonate from northeastern Thailand presenting with neonatal anemia (Hb level 7.7 g/dL, Hb H 1.5%, and Hb Bart’s 29.4%) [17]. The present study documents a case of Hb H Chumphae from southern Thailand for the first time. The 7-year-old girl had clinical hemolytic anemia and neonatal jaundice since birth and received a diagnosis of unknown Hb H disease. Hb analysis at birth revealed Hb Bart’s 31.3%, Hb A 19.5%, and Hb F 44.5%. Rather than decreasing, the Hb Bart’s level increased commensurately with the increasing number of defective α-globin genes as the patient progressed beyond infancy. During the study, the girl had asymptomatic mild anemia with no history of blood transfusion. The clinical phenotype appeared milder with increasing age, suggesting a potential improvement in clinical severity. However, the patient still showed an unusually high Hb Bart’s level at 25.9%, with Hb H level at 0.8%. This Hb Bart’s level was notably higher compared to that in other non-deletional Hb H diseases. For example, individuals with Hb H-CS disease typically present with a median Hb Bart’s level of 5.5% (IQR: 3.1–7.8%) (Table 2). This unusual finding suggests that Hb Chumphae may co-migrate with Hb Bart’s at zone 12, causing an artificially elevated Hb Bart’s level when measured using the CE technique (Figure 4). A recent study supported our hypothesis by demonstrating that Hb Chumphae could be clearly separated from Hb Bart’s when using two automated Hb analyzers, the Premier Resolution and Bio-Rad Variant II systems [20], confirming that the CE technique was responsible for the co-migration of these two hemoglobin fractions. Therefore, when a patient with non-deletional Hb H disease has a higher Hb Bart’s level than expected, particularly when using the CE technique, further laboratory investigation should be performed to evaluate the possibility of Hb H-Chumphae disease. The CE technique is the most widely used method in Thailand but may not accurately distinguish Hb Chumphae from Hb Bart’s, leading to falsely elevated levels of Hb Bart’s.

**Figure 5:**
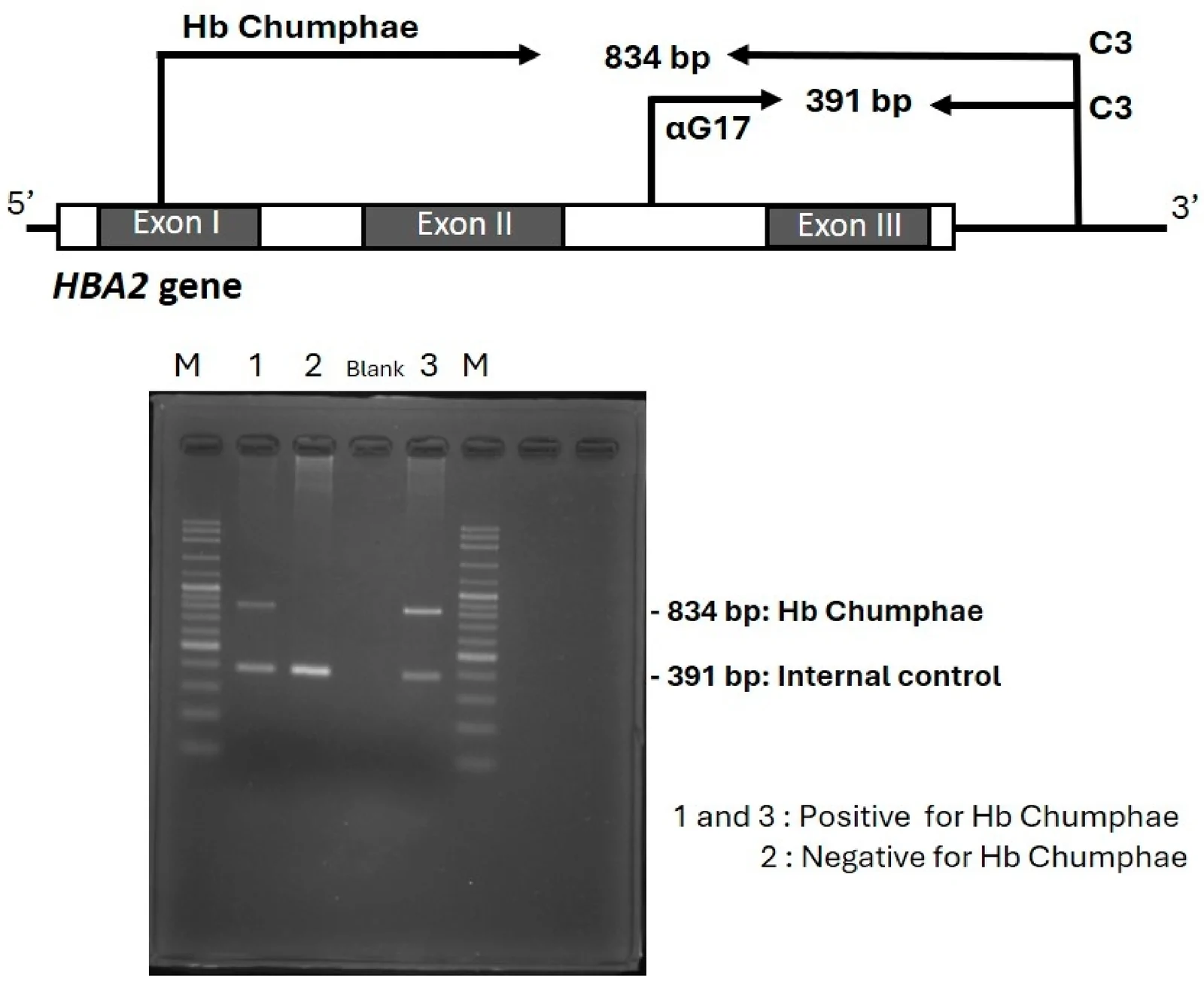
Schematic diagram of primer orientation and agarose gel electrophoresis results for a newly developed AS-PCR of Hb Chumphae.

Furthermore, this study provides the first description of a 50-year-old male patient homozygous for Hb Chumphae, confirmed via MLPA and DNA sequencing. However, we could not contact the patient to investigate the possibility of consanguineous marriage as an underlying cause of this rare homozygous genotype. The presence of a 5.5% peak in zone 12 (the Hb Bart’s zone) on CE is a key indicator for additional molecular testing to identify the presence of the Hb Chumphae variant. Screening for the Hb Chumphae variant among Thai individuals with possible carriers of α-thalassemia mutations yielded no positive results, suggesting its rarity in the Thai population. Therefore, further studies with larger samples are warranted to elucidate the prevalence and frequency of Hb Chumphae across Thailand. The rapid AS-PCR developed in the present study will be beneficial to future research (Figure 5).

The degree of disproportion between α- and non-α-globin chains influences the clinical severity of hemoglobin disorders. In Hb E/β-thalassemia disease, coinheritance of α^0^-thalassemia ameliorates clinical severity [21,22], as loss of two out of the four functional *HBA* genes reduces excess α-globin chain production, thereby partially correcting the globin chain imbalance. In contrast, the coinheritance of Hb E and non-deletional Hb H disease appears insufficient to reduce the level of α- and non-α-globin chain disproportion. This result can be explained by non-deletional Hb H disease, which results from mutations affecting three of the four *HBA* genes, leaving only a single functional *HBA* gene and leading to severely decreased α-globin chain production. Moreover, Hb E is categorized as a β^+^-thalassemia mutation due to aberrant mRNA splicing, which reduces β^E^-globin output by approximately 25–35% in Hb E heterozygotes [19]. Therefore, the modest reduction in β-globin synthesis conferred by Hb E heterozygosity is inadequate to offset the profound α-globin deficit already present in non-deletional Hb H disease. Thus, hemoglobin levels did not differ significantly between NTDT patients with solely non-deletional Hb H disease alone and those with co-inherited Hb E heterozygosity. However, patients with non-deletional Hb H disease co-inherited with Hb E heterozygosity had significantly lower MCV and MCH values compared to those with non-deletional Hb H disease alone. This finding can be attributed to the additional reduction in β-globin synthesis imposed by the Hb E variant, which further decreases the total number of functional globin chains available for hemoglobin assembly within each erythrocyte. As a result, the hemoglobin quantity per red blood cell is further compromised beyond that observed in non-deletional Hb H disease alone, leading to more pronounced microcytosis and hypochromia as reflected by the lower MCV and MCH values. The hematological parameters observed in our cohort for non-deletional Hb H disease, both alone and with co-inherited Hb E heterozygosity, are consistent with previous reports [15,17,23]. However, these results contrast with those of a study that reported a higher proportion of non-deletional Hb H patients co-inherited with Hb E heterozygosity requiring more regular blood transfusions than those without Hb E heterozygosity co-inheritance. Furthermore, among NTDT patients, individuals with co-inheritance of non-deletional Hb H disease and heterozygous Hb E exhibited significantly lower baseline Hb levels (median 8.1, IQR 7.2–8.6 g/dL) compared to those without co-inherited Hb E (median 8.8, IQR 8.2–9.5 g/dL; *p* < 0.001). Additionally, a significantly greater proportion of patients with non-deletional Hb H disease and co-inherited heterozygous or homozygous Hb E experienced acute hemolytic events at presentation (50.5%) compared to those with non-deletional Hb H disease alone (34.8%; *p* = 0.015) [24]. Nevertheless, further research with larger samples and diverse ethnic backgrounds is warranted to fully elucidate these relationships and identify modifying factors.

In Hb E heterozygotes without α-thalassemia, Hb E typically accounts for approximately 25–35%. Coinheritance of α-thalassemia lowers this proportion in relation to the number of functional α-globin genes affected [25]. Non-deletional Hb H disease co-inherited with Hb E heterozygosity characteristically presents with a reduced proportion of Hb E (approximately 13–15%), with a notable absence of Hb H and trace amounts of Hb Bart’s [19]. In the present case, the patient carrying non-deletional Hb H disease (α1 IVS1-1 (G>A)) and Hb E heterozygosity demonstrated a hemoglobin profile consistent with Hb EA pattern, with a Hb E level of 15.7% and complete absence of both Hb H and Hb Bart’s. This hemoglobin profile closely resembles that of Hb E heterozygote, posing a significant risk of misdiagnosis. Nevertheless, the disproportionately reduced Hb E level (as low as 15.7%) serves as a critical diagnostic clue, reflecting a markedly diminished availability of α-globin chains. This reduction can be attributed to the lower binding affinity of α-globin chains for β^E^-globin compared with β^A^-globin chains, which consequently results in a lower proportion of Hb E in individuals co-inheriting Hb E heterozygosity with Hb H disease than in those with Hb E heterozygosity alone.

The present study revealed the presence of Hb Adana in southern Thailand, a region in close geographical proximity to neighboring countries such as Malaysia and Indonesia, where this variant is known to occur at a high frequency [26,27]. Hb Adana was first reported in 1993 in two Turkish patients with compound heterozygous Hb Adana /α^0^-thalassemia (–(α)^20.5^, NG_000006.1:g.15164_37864del22701)) who presented with severe clinical manifestations [28]. Hb Adana is a highly unstable variant, and the complex interactions between Hb Adana and other α-thalassemia variants may give rise to a broad spectrum of clinically significant conditions, including Hb H disease, Hb H-Hydrops disease, and Hb Bart’s hydrops fetalis [26,29,30]. Notably, three cases of Hb H-like disease resulting from the compound heterozygous interaction of Hb Adana with Hb CS are reported for the first time in southern Thailand in this study. From a diagnostic perspective, Hb Adana is undetectable by either CE or high-performance liquid chromatography in both carriers and individuals with compound heterozygosity. However, in cases of compound heterozygous Hb Adana and Hb CS, discordance among hematological parameters, Hb analysis findings (such as the presence of Hb Bart’s or Hb H), and routine DNA analysis results revealing only a single *HBA* gene abnormality should prompt DNA sequencing to identify additional mutations. Therefore, PCR-based molecular diagnostic methods capable of detecting non-deletional α^T^-thalassemia variants are essential for accurate diagnosis. Accordingly, we incorporated a specific primer for Hb Adana detection into a previously established AS-PCR for identifying Hb CS and Hb PS [31], resulting in a newly developed multiplex AS-PCR capable of simultaneously identifying single mutation and their interactions among the three non-deletional α^T^-thalassemia variants (Figure 3). However, this multiplex AS-PCR can detect the Hb Adana variant only in the *HBA2* gene but not in the *HBA1* gene. Nevertheless, evidence across various populations consistently indicates that the Hb Adana variant occurs more frequently on *HBA2* than on *HBA1* [30], and all the three patients in the present study were confirmed to carry the Hb Adana variant on *HBA2*. This distinction is clinically relevant, as homozygosity or compound heterozygosity involving Hb Adana on *HBA2* with other α-thalassemia mutations has been associated with severe clinical outcomes, such as Hb H-hydrops fetalis [32,33]. In contrast, homozygosity for the Hb Adana on *HBA1* typically presents with only mild microcytic anemia [34]. Collectively, these findings support the implementation of this newly developed multiplex AS-PCR as a routine diagnostic tool for severe α-thalassemia in Southeast Asian region.

In this study, α-thalassemia mutations were identified in 525 of 679 individuals with possible α-thalassemia carriers (77.3%). In the remaining 154 individuals (22.7%), microcytosis may be explained by iron deficiency, which is prevalent in Thailand [35]. Nevertheless, iron studies were not performed in all samples, which is a limitation of this study.

In conclusion, this study highlighted the genetic heterogeneity of non-deletional Hb H and Hb H-like diseases in the southern Thai population. We reported the first case of Hb CS compound heterozygosity with Hb Adana and homozygous Hb Chumphae in the Thai population. The updated data on α-thalassemia allele proportions among screened carriers support the consideration of including Hb Adana detection in diagnostic panels for Southern Thailand, particularly in appropriate clinical or hematological centers. Furthermore, studies with larger and more representative samples are needed to confirm its regional relevance to expedite accurate thalassemia diagnosis.

## 4. Methods

The study protocol was approved by the Human Research Ethics Unit of the Faculty of Medicine, Prince of Songkla University (REC 64-054-5-2). Due to our use of unidentifiable leftover specimens, the Human Research Ethics Unit waived the requirement for informed consent. Laboratory procedures strictly adhered to the Thai national guidelines for identifying thalassemia and hemoglobinopathy.

### 4.1. Samples

A total of 118 leftover DNA samples from patients with non-deletional Hb H or Hb H-like diseases were obtained from the thalassemia unit and the Department of Pathology, Faculty of Medicine, Prince of Songkla University, Hatyai, Thailand. Furthermore, 679 DNA samples were included from individuals with possible α-thalassemia carriers based on first-level hematology screening, with MCV < 80 fL and Hb analysis showing a pattern A2A with HbA2 < 3.5% from three institutes across Thailand. Samples that met the above-mentioned criteria underwent molecular analysis regardless of iron status. Individuals with an abnormal Hb fraction, including Hb H or Hb Bart’s, were excluded. The MCH was not used as an inclusion criterion in this study. Possible α-thalassemia carrier samples were obtained from 270 subjects from the School of Allied Health Science, University of Phayao (north); 200 subjects from the Department of Pathology, Faculty of Medicine, Maha Chakri Sirindhorn Medical Center, Srinakharinwirot University (central and east); and 209 subjects from the Department of Pathology, Faculty of Medicine, Prince of Songkla University (south).

### 4.2. Hematological Analysis

Each center conducted complete blood count and Hb analysis. Hematological parameters were determined using an automated blood cell counter (Sysmex XN 3000; Sysmex, Kobe, Japan), whereas Hb analysis was conducted via capillary electrophoresis (CE) using the CAPILLARYS 2 system (Sebia, Lisses, France). Blood transfusion history of patients with non-deletional Hb H disease was reviewed, and hematologic parameters were measured at steady state and in patients with NTDT and before blood transfusion in patients with TDT. No measurements were obtained during a hemolytic crisis.

### 4.3. Molecular Diagnosis of α-Thalassemia

α-thalassemia mutations, including – –SEA, – –THAI, – –CR, – –SA, – –FIL (NG_000006.1:g.11684_43534del31851), – –MED (NG_000006.1:g.24664_41064del16401), –(α)^20.5^, –α^3.7^, –α^4.2^, Hb CS, and Hb PS, were identified using polymerase chain reaction (PCR)-based methods, as previously described [12,31].

### 4.4. DNA Sequencing and Multiplex Ligation-Dependent Probe Amplification (MLPA) Analysis

Patients with non-deletional Hb H with uncharacterized genotypes and individuals who are possible α-thalassemia carriers based on first-level hematology screening with unidentified mutations were further screened for non-deletional α-thalassemia mutations via *HBA* sequencing using an ABI PRISM™ 3130 XL analyzer (Applied Biosystems, Foster City, USA) or barcode-tagged sequencing based on the next-generation sequencing technique.

MLPA for large deletions detection in the HBA cluster was performed using the commercial SALSA MLPA (P140-C1 *HBA* probemix) kit (MRC-Holland, Amsterdam, The Netherlands). The reaction was run on a SimpliAmp™ thermal cycler (Thermo Fisher Scientific, Waltham, USA) following the manufacturer’s instructions. Subsequent fragment analysis was performed using an ABI PRISM™ 3500 analyzer (Applied Biosystems, Foster City, CA, USA), and data were interpreted using the Coffalyser.Net™ software version 650513.1733 (MRC Holland).

### 4.5. Development of Multiplex Allele-Specific PCR (AS-PCR) for Detection of Hb CS, Hb PS, and Hb Adana

A multiplex allele-specific PCR (AS-PCR) was developed for rapid screening of three non-deletional α^T^-thalassemia variants—Hb CS, Hb PS, and Hb Adana. In this simultaneous detection system, primers αG17 and C3, which generate a 391 bp fragment, were used as an internal control. Three specific primer pairs—αG2 and C3, αG17 and αG18 [31], and Hb Adana and C3—were used to detect Hb CS (180 bp), Hb PS (253 bp), and Hb Adana (569 bp), respectively (Table 5 and Figure 3).

The PCR mixture (total volume, 25 µL) contained 50–200 ng of genomic DNA, 0.16 pmole of primer αG17, 0.32 pmole of primer αG18, 0.4 pmole of primer αG2 and Hb Adana, 0.48 pmole of primer C3, 200 µM of each dNTPs, 1.75 mM MgCl_2_, 1 M Betaine, 1.25% DMSO, 0.5 U of Taq DNA polymerase (Vivantis Technologies, Subang Jaya, Malaysia), 16 mM (NH_4_)_2_SO_4_, 50 mM Tris-HCl (pH 9.2) buffer, and 0.1% Triton^TM^ X-100. The amplification reaction was performed on a SimpliAmp^TM^ thermal cycler (Thermo Fisher Scientific, Waltham, USA). After initial heating at 95 °C for 10 min, the reaction was followed by 30 cycles of 95 °C for 60 s, 62 °C for 60 s, and 72 °C for 60 s and a final extension at 72 °C for 5 min. The amplified products were separated by 1.5% agarose gel electrophoresis and visualized under ultraviolet light after ethidium bromide staining.

### 4.6. Development of AS-PCR for the Detection of Hb Chumphae

A 391 bp fragment generated using primers αG17 and C3 [13] was used as an internal control. An 834 bp fragment specific to the Hb Chumphae variant was amplified using primer Hb Chumphae and primer C3 (Table 5 and Figure 5). The PCR mixture (25 μL) contained 50–200 ng of genomic DNA, 0.4 pmoles of primer C3, 0.16 pmoles of primer αG17, 0.32 pmoles of primer Hb Chumphae, 200 μM of dNTPs, 1 M Betaine, 1.5 mM MgCl_2_, 1.25% DMSO, 0.5 unit of Taq DNA polymerase (Vivantis Technologies, Subang Jaya, Malaysia), 50 mM KCl, 10 mM Tris-HCl (pH 9.1) buffer, and 0.1% Triton^TM^ X-100. The amplicon was amplified with initial heating at 95 °C for 5 min, followed by 30 cycles of 95 °C for 1 min, 63 °C for 45 s, and 72 °C for 1 s and final extension at 72 °C for 10 min on a SimpliAmp^TM^ thermal cycler (Thermo Fisher Scientific, Waltham, USA). The PCR products were separated via 1.5 agarose gel electrophoresis for 30 min and visualized under ultraviolet light after ethidium bromide staining.

### 4.7. Statistical Analysis

The hematological parameters of two independent groups were compared using the Mann–Whitney *U* test in Minitab (version 14.0, Minitab Inc.). A *p*-value < 0.05 was considered statistically significant.

## Figures and Tables

**Table 1:** Point mutations identified in the *HBA2*/*HBA1* genes and their effects on function or stability of hemoglobin molecules.

α^T^-Thalassemia Variants (Common Name)	Gene	Position	Nucleotide Change (HGVS)	Amino Acid Change	Effect/Stability
Initiation-codon (-A)	*HBA2*	Initiation codon ATG>-TG	c.1delA	Met>0	No translation
Hb Chumphae	*HBA2*	Codon 10 GTC>GAC	c.32T>A	Val>Asp	Unstable
α2 codon 30 (-GAG)	*HBA2*	Codon 30 -GAG	c.91_93del	Glu>0	Unstable
Hb Adana	*HBA2*	Codon 59 GGC>GAC	c.179G>A	Gly>Asp	Unstable
Hb Quong Sze	*HBA2*	Codon 125 CTG>CCG	c.377T>C	Leu>Pro	Unstable
Hb Constant Spring	*HBA2*	Codon 142 TAA>CAA	c.427T>C	Term>Gln	Unstable
α1 IVS1-1 (G>A)	*HBA1*	IVS1-1 G>A	c.95+1G>A	-	No mRNA splicing
Hb Queens Park	*HBA1*	Codon 32 ATG>AAG	c.98T>A	Met>Lys	Unstable

Abbreviations: Met, methionine; Val, valine; Asp, aspartic acid; Glu, glutamic acid; Gly, glycine; Leu, leucine; Pro, proline; Gln, glutamine; Lys, lysine; Term, termination (stop) codon; del, deletion; Hb, hemoglobin; HGVS, Human Genome Variation Society; IVS, intervening sequence (intron).

**Table 2:** Molecular spectrum and hematological parameters of 118 patients with non-deletional Hb H (compound heterozygosity for α^0^-thalassemia and α^T^-thalassemia) and Hb H-like disease (genotypes involving two α^T^-thalassemia alleles, α^T^-thalassemia/α^T^-thalassemia).

Thalassemia Disease	Genotype	Frequency	*n*	Hb	Hct	MCV	MCH	RDW	Hb CS	Hb H	Hb Bart’s	Hb A_2_	Hb A_2_/E
		(%)		(g/dL)	(%)	(fL)	(pg)	(%)	(%)	(%)	(%)	(%)	(%)
Hb H-CS	– –^SEA^/α^CS^α	79.7	94	7.5 (6.2–8.5)	27.3 (24.1–30.3)	71.0 (65.5–77.5)	19.2 (18.0–20.3)	25.6 (23.2–27.6)	1.9 (1.3–2.3)	11.9 (7.9–16.7)	5.5 (3.1–7.8)	1.3 (0.9–1.9)	
Hb H-CS with Hb E heterozygosity	– –^SEA^/α^CS^α, β^E^/β	11.9	14	7.1 (6.6–7.9)	25.3 (23.0–27.9)	56.0 (53.8–60.0)	16.1 (15.1–16.8)	26.0 (24.2–29.3)	1.3 (0.9–1.6)	0.0	3.9 (1.4–6.9)		13.8 (12.2–15.3)
Hb H-α1 IVSI-1 (G>A) with Hb E heterozygosity	– –^SEA^/αα^IVS I-1^, β^E^/β	0.8	1	9.6	32.0	55.0	19.4	22.8	0.0	0.0	0.0		15.7
Hb H-Quong Sze with Hb E heterozygosity	– –^SEA^/α^QS^α, β^E^/β	0.8	1	8.8	29.1	51.8	15.7	37.0	0.0	0.0	0.3		9.4
Hb H-α2 Int (-A)	– –^THAI^/α^Int (-A)^α	0.8	1	6.7	23.2	59.0	16.9	17.7	0.0	15.3	0.0	1.5	
Hb H-α2 codon 30 (-GAG)	– –^SEA^/α^CD30^α	0.8	1	7.1	27.1	78.0	21.3	29.2	0.0	13.6	7.0	0.7	
Hb H-Queens Park	– –^SEA^/αα^QP^	0.8	1	8.5	29.7	52.8	15.2	23.4	0.0	3.0	0.0	2.3	
Hb H-Chumphae	– –^SEA^/α^CP^α	0.8	1	10.1	36.8	71.7	19.7	33.7	0.0	0.8	25.9	1.3	
Homozygous Hb Chumphae	α^CP^α/α^CP^α	0.8	1	10.6	36.7	65.5	17.9	23.0	0.0	0.0	5.5	1.6	
Hb CS with Hb Adana	α^CS^α/α^Adana^α	2.5	3	7.2 (6.7–8.7)	29.8 (24.7–30.9)	101.7 (77.0–124.1)	28.2 (22.5–28.9)	27.0 (23.3–29.0)	1.0 (0.9–4.9)	0.0 (0.0–4.0)	0.8 (0.0–1.1)	1.9 (1.1–3.2)	

Data are presented as median (interquartile range) or raw data as appropriate. Genotypes combining α^0^-thalassemia with α^T^-thalassemia variants are compound deletional/non-deletional α-thalassemia. Hematologic parameters represent values at steady state in NTDT patients or values before blood transfusion in TDT patients.. Abbreviations: CS, Constant Spring; Hb, hemoglobin; Hct, hematocrit; Int, Initiation codon; MCH, mean corpuscular hemoglobin; MCV, mean corpuscular volume; RDW, red cell distribution width.

**Table 3:** Comparison of hematological parameters between non-deletional Hb H disease and non-deletional Hb H disease combined with Hb E heterozygosity in patients with non-transfusion-dependent thalassemia (NTDT).

Parameter	Non-Deletional Hb H Disease		Non-Deletional Hb H Disease with Hb E Heterozygosity		*p* Value
	Median	(Interquartile range)	Median	(Interquartile range)	
NTDT (*n*, %)	75/98 (76.5%)		11/16 (68.8%)		0.5090
Hb (g/dL)	8.0	(7.0–8.7)	7.7	(7.0–9.2)	0.9054
Hct (%)	28.2	(25.6–31.0)	26.8	(24.7–31.0)	0.4136
MCV (fL)	71.0	(65.0–76.0)	56.0	(54.0–60.0)	0.0001
MCH (pg)	18.9	(17.8–20.0)	16.7	(15.6–17.2)	0.0003
RDW (%)	25.3	(23.0–27.3)	25.1	(23.4–27.8)	0.9190

Abbreviations: Hb, hemoglobin; Hct, hematocrit; MCH, mean corpuscular hemoglobin; MCV, mean corpuscular volume; RDW, red cell distribution width.

**Table 4:** Proportions of each variant among the detected α-thalassemia alleles in 525 (77.3%) cases, out of 679 possible α-thalassemia carriers based on first-level hematology screening, across three reference laboratory centers in Thailand.

α-Thalassemia	Northern	Proportion	Central and Eastern	Proportion	Southern	Proportion	Three Centers	Proportion
	(*n* = 259)	(%)	(*n* = 151)	(%)	(*n* = 163)	(%)	(*n* = 573)	
– –SEA	162	62.5	49	32.5	69	42.3	280	48.9
– –THAI	2	0.8	1	0.7	0	0.0	3	0.5
–α^3.7^	65	25.1	92	60.9	85	52.1	242	42.2
–α^4.2^	7	2.7	4	2.6	1	0.6	12	2.1
Hb Constant Spring	23	8.9	4	2.6	5	3.1	32	5.6
Hb Paksé	0	0.0	1	0.7	0	0.0	1	0.2
Hb Adana	0	0.0	0	0.0	3	1.8	3	0.5
Hb Chumphae	0	0.0	0	0.0	0	0.0	0	0.0

n; represents number of mutant alleles; 7 samples with compound heterozygotes α^+^-thalassemia/α^T^-thalassemia (α^CS^α/–α^3.7^, n = 6; α^PS^α/–α^3.7^, n = 1), and 41 samples with homozygotes α+-thalassemia (–α^3.7^/–α^3.7^, n = 37; –α^4.2^/–α^4.2^, n =1; –α^3.7^/–α^4.2^ n = 3), and no extra Hb fraction (e.g., Hb H or Hb Bart’s) was detected in cases with compound heterozygosity for α^+^-thalassemia/α^T^-thalassemia. Abbreviations: Hb, hemoglobin.

**Table 5:** Sequences and the characteristics of the oligonucleotide primers used for multiplex AS-PCR for Hb Constant Spring, Hb Paksé, and Hb Adana and monoplex AS-PCR for Hb Chumphae.

Primer Name	Targeted Variants	Sequence 5′-3′	Oligo Length (bp)	%GC	Tm (°C)	Reference
C3	Internal control	CCATTGTTGGCACATTCCGGGATA	24	50.0	64.7	31
αG2	Hb Constant Spring	GCTGACCTCCAAATACCGT**C**	20	55.0	60.8	31
αG17	Internal control	AGATGGCGCCTTCCTCTCAGG	21	61.9	65.3	31
αG18	Hb Paksé	ACGGCTACCGAGGCTCCAGC**A**	21	67.0	68.5	31
Hb Adana	Hb Adana	CCCAGGTTAAGGGCCACG**A**	19	63.2	61.7	This study
Hb Chumphae	Hb Chumphae	CCTGCCGACAAGACCAACG**A**	20	60.0	65.6	This study

Oligonucleotide bases specific to the targeted variants are shown in bold. Abbreviations: AS-PCR, allele-specific polymerase chain reaction; Hb, hemoglobin; Tm, melting temperature.

## Data Availability

The datasets generated and/or analyzed in the current study are available from the corresponding author upon reasonable request.
